# Dopamine Inactivation Efficacy Related to Functional DAT1 and COMT Variants Influences Motor Response Evaluation

**DOI:** 10.1371/journal.pone.0037814

**Published:** 2012-05-23

**Authors:** Stephan Bender, Thomas Rellum, Christine Freitag, Franz Resch, Marcella Rietschel, Jens Treutlein, Christine Jennen-Steinmetz, Daniel Brandeis, Tobias Banaschewski, Manfred Laucht

**Affiliations:** 1 Section for Clinical Neurophysiology and Multimodal Neuroimaging, Child and Adolescent Psychiatric Department, Technical University Dresden, Dresden, Germany; 2 Department of Child and Adolescent Psychiatry, Psychosomatics and Psychotherapy, Johann-Wolfgang-Goethe University, Frankfurt am Main, Germany; 3 Department of Child and Adolescent Psychiatry and Psychotherapy, Heidelberg University Hospital, Heidelberg, Germany; 4 Department of Child and Adolescent Psychiatry and Psychotherapy, Central Institute of Mental Health, Medical Faculty Mannheim/Heidelberg University, Mannheim, Germany; 5 Department of Genetic Epidemiology in Psychiatry, Central Institute of Mental Health, Medical Faculty Mannheim/Heidelberg University, Mannheim, Germany; 6 Department of Biostatistics, Central Institute of Mental Health, Medical Faculty Mannheim/Heidelberg University, Mannheim, Germany; 7 Department of Child and Adolescent Psychiatry, University of Zürich, Zürich, Switzerland; 8 Center for Integrative Human Physiology, University of Zürich, Zürich, Switzerland; 9 Department of Psychology, University of Potsdam, Potsdam, Germany; Radboud University, Netherlands

## Abstract

**Background:**

Dopamine plays an important role in orienting, response anticipation and movement evaluation. Thus, we examined the influence of functional variants related to dopamine inactivation in the dopamine transporter (*DAT1*) and catechol-O-methyltransferase genes (*COMT*) on the time-course of motor processing in a contingent negative variation (CNV) task.

**Methods:**

64-channel EEG recordings were obtained from 195 healthy adolescents of a community-based sample during a continuous performance task (A-X version). Early and late CNV as well as motor postimperative negative variation were assessed. Adolescents were genotyped for the *COMT* Val^158^Met and two *DAT1* polymorphisms (variable number tandem repeats in the 3′-untranslated region and in intron 8).

**Results:**

The results revealed a significant interaction between *COMT* and *DAT1*, indicating that *COMT* exerted stronger effects on lateralized motor post-processing (centro-parietal motor postimperative negative variation) in homozygous carriers of a *DAT1* haplotype increasing DAT1 expression. Source analysis showed that the time interval 500–1000 ms after the motor response was specifically affected in contrast to preceding movement anticipation and programming stages, which were not altered.

**Conclusions:**

Motor slow negative waves allow the genomic imaging of dopamine inactivation effects on cortical motor post-processing during response evaluation. This is the first report to point towards epistatic effects in the motor system during response evaluation, i.e. during the post-processing of an already executed movement rather than during movement programming.

## Introduction

The perception-action-cycle involves different stages, from the orienting to a warning stimulus to the preparation of a reaction movement and the evaluation of the given response. Due to the high time resolution of electroencephalography, these stages can be excellently assessed in contingent negative variation (CNV) paradigms, which involve a cue followed by a later imperative stimulus which requires a motor response [1]. The early component of the CNV reflects an orienting response and early response preparation [2], [3], [4]. The late component of CNV reflects the preparation of the motor response and the anticipation of the imperative stimulus [5], [6]. Postimperative negative variation (PINV) refers to motor and cognitive response evaluation and may include a short-term memory trace of the planned movement which needs to be compared to reafferent sensory feedback about the actually performed movement [7], [8], [9]. Dopamine modulates the neuronal signal-to-noise ratio in order to focus prefrontal cortical resources [10] and plays an important role in focusing attention on relevant stimulus characteristics [11]. Thus, dopamine plays an important role in all three stages, orienting [12], response preparation [13] and response evaluation [14]. However, it has not been examined yet, how specific genetic variations affect the different cognitive and motor processing stages:

The duration of dopaminergic action is limited by dopamine inactivation, i.e. mainly methylation by catechol-O-methyltransferase (COMT) in the prefrontal cortex [15] and reuptake via the dopamine transporter (DAT1) in the striatum [16].

In the current study, we investigated the effects of three functional polymorphisms in the *COMT* and *DAT1* genes on the orientation reaction, movement programming and stimulus post-processing (indexed by the early and late components of the CNV as well as the motor postimperative negative variation component), during a continuous performance test with speeded button press response movements.

A widely studied functional *COMT* polymorphism, characterized by the substitution of valine for methionine at codon 158 [17], results in less enzyme activity and higher extracellular dopamine levels [18]. The 10-repeat allele of a variable number tandem repeat (VNTR) polymorphism in the 3′-untranslated-region of *DAT1* and the 6-repeat allele of a VNTR in intron 8 lead to greater *DAT1* expression [19] and reduced striatal dopamine levels, though the controversy about whether the 10-repeat allele results in greater or lower *DAT* expression [20] is not finally resolved yet. The co-occurrence of both *DAT1*-expression increasing VNTRs, the 6R–10R haplotype, has been reported to strengthen this effect [21], [22]. Thus, here we examined the *DAT1* haplotype and its interaction with the *COMT* Val^158^Met polymorphism in relation to motor PINV.

Previous fMRI genomic imaging studies have demonstrated that higher prefrontal (Met *COMT* allele [23], [24], [25]) and lower striatal (10R *DAT1* allele [24]) dopamine levels resulted in more focused prefrontal cortical activation.

We hypothesized that motor CNV component amplitudes would be genetically affected in a similar way to prefrontal cortex BOLD responses in working memory tasks [24]. In this case, the Met-*COMT* allele and the 6R–10R *DAT1* haplotype would be associated with more focused motor activation with lower amplitudes. An alternative hypothesis was that *DAT1* and *COMT* effects on motor CNV components would show exactly the inverse pattern because more prefrontal resources could be required in prefrontal-motor loops to act on less intense motor representations [26]. Because *DAT1* and *COMT* both affect the *termination* of dopaminergic neurotransmission, we hypothesized that motor PINV would be more strongly affected than early CNV or the initial motor potential peak, because the duration and amplitude of motor post-processing during motor PINV could depend on the speed of inactivation of dopamine released during the preceding response. Even if this specific hypothesis would be falsified, the assessment of the two functional genetic polymorphisms would allow an examination of dopaminergic effects on reaction-related EEG potentials in healthy adolescents.

## Methods

### Participants

The current data analysis was conducted on the sample of the Mannheim Study of Children at Risk, a prospective longitudinal study of the outcome of early risk factors from infancy into adulthood [22]. Children born between 1986 and 1988 were recruited from two obstetric and six pediatric hospitals of the Rhine-Neckar Region of Germany. Infants were included consecutively into the study according to a 2-factorial design intended to enrich and to control the risk status of the sample (full details of the sampling procedure have been reported previously [27]). As a result, approximately two thirds of the study sample had experienced obstetric complications such as preterm birth, and about two thirds of the families had psychosocial adversities such as marital discord or chronic difficulties. Of the initial sample of 384 participants, 18 (4.7%) were excluded because of severe handicaps (neurological disorder, intelligence quotient <70 or motor quotient <70), 28 (7.3%) were drop-outs at age 15, 35 (9.1%) refused to take part in blood sampling or had incomplete genetic data, and from 43 (11.2%), no, or no reliable, EEG data were available. Intelligence was assessed at the age of 11 years using the Culture Fair Test 20 [28], [29]; the motor quotient was determined at age 11 years by a short version of the Body Coordination Test for children KTK [30]. 65 subjects (16.7%) were excluded from the current analysis due to a current psychiatric DSM-IV diagnosis. 21 subjects of the remaining 195 (10.8%) had to be excluded because they were not right-handed as indicated by a handedness index above +60 in the Edinburgh Handedness Inventory [31]. All subjects were free of psychoactive medication at the time of the recording. The study was approved by the ethics committee of the Medical Faculty of the University of Heidelberg/Mannheim. Written informed consent was obtained from all participants and their parents. All subjects had a corrected visual acuity of 0.8 or higher.

### Recordings

Continuous 64-channel DC EEG was recorded by Neuroscan Sympamps amplifiers (Neuroscan Inc., TX, USA). Sintered silver/silver chloride electrodes were positioned by an equidistant electrode cap (Easycap, FMS, Germany). Electrode impedances were kept below 10 kOhm. Vertical electrooculogram (VEOG) was recorded by electrodes 1 cm below and above the left eye. Horizontal electrooculogram (HEOG) was calculated by leads F9′ and F10′ next to the outer canthi. Small deviations of electrode positions from the international 10-10 system are indicated by apostrophes. The recording reference was placed near the left mastoid. Offline, data were transformed to average reference. The sampling rate was 500 Hz. An anti-aliasing low-pass filter with a cut-off frequency of 100 Hz was employed. The visual stimulation was presented by Gentask of the Neuroscan Stim software package. Reaction times were collected from response triggers from the response pad.

### Task

Subjects performed a computerized A-X version of the continuous performance test (CPT; constructed by doubling the number of trials of a common previous multicenter version [32], [33], [34]). 800 black-colored capital letters were presented on white background in the center of the computer screen for 150 ms. The stimulus onset asynchrony (SOA) between the different letters was 1600 ms. Whenever an ‘A’ was followed by an ‘X’ (50% probability), subjects had to respond with a fast right-hand button press with their index finger on the response pad. The ‘A’ was followed by an ‘X’ 80 times and by another letter 80 times. Additionally, single distractor letters were presented.

An ‘X’ without a preceding ‘A’ occurred 80 times. Another nine letters of the alphabet (‘B’, ‘C’, ‘D’, ‘E’, ‘F’, ‘G’, ‘H’, ‘J’, ‘L’) were employed as distractors. The distractor ‘H’ occurred 160 times (frequent distractor). The distractors ‘B’, ‘C’, ‘D’, ‘E’, ‘F’, ‘G’, ‘J’, and ‘L’ appeared 40 times each.

### Data pre-processing

For the analysis of early and late CNV, continuous recordings were segmented into stimulus-locked segments from 400 ms prior to the distractor before the warning stimulus ‘A’ to 1600 ms after the imperative stimulus ‘X’ (5.2 seconds in total). The 400 ms before the warning stimulus ‘A’ served as baseline.

For the analysis of the initial motor potential peak during movement execution and the analysis of motor post-processing, response-locked epochs of 4 seconds were created, beginning 2800 ms before the response until 1200 ms afterwards. The first 400 ms of this interval served as baseline (2800 to 2400 ms before the response). Only trials with correct responses within 800 ms were included in the analysis. Taking into account the stimulus onset asynchrony of 1600 ms, this assured that the baseline interval was situated before the onset of the warning letter ‘A’ even for slow responses. The warning letter ‘A’ of a target sequence was never directly preceded by another target sequence. Thus, during the baseline, the subjects had processed a distractor letter and were waiting for the next stimulus to occur. Reaction times took at least approximately 150–200 ms, even for fast responses. We verified that there were no gene effects on the baseline time interval which could have influenced the results.

Data were corrected for eye movements and blinks by the algorithm of Gratton and Coles (Brain Vision Analyzer, BrainProducts GmbH, Munich, Germany). Average reference was calculated offline. Data were 30 Hz low-pass filtered by a zero-phase shift Butterworth filter with a slope of 48 dB/octave. Potentials exceeding 150 µV amplitude were rejected automatically as artifacts; remaining smaller artifacts were removed by an experienced EEG technician who was blind to the study hypotheses.

We calculated lateralized movement-related potentials by subtracting the potentials at each electrode on the right hemisphere from the potentials measured at its homologue on the left side of the head (e.g. C3–C4). In this way, the resulting polarity reflects the polarity for potentials which were lateralized contralateral to the response movement with the right hand. In a previous study, we had found that lateralization in trials with the dominant right hand closely resembled the final lateralized movement-related potential [9]. A complete calculation of the lateralized movement-related potential to eliminate all stimulus-related lateralized potentials was not possible because only responses by the right hand were available. However, due to a mean reaction time of about 350 ms and a motor PINV interval 400–800 ms after the response, summing up to a window 750–1150 ms after the imperative stimulus ‘X’ which was presented in the center of the visual field, there should be only very small influences of lateralized stimulus-related processes.

### Data analysis

#### Early CNV (orienting reaction)

Early CNV amplitude was measured at its topographical maximum at Fz during the time interval 600–900 ms [2], [35].

#### Late CNV (movement preparation and programming)

The amplitude of the motor part of late CNV amplitude was determined as the mean potential 200 ms before the imperative stimulus (‘X’) over the left (contralateral to the response movement) motor (pooled leads C3, CP3′, C5′) and the supplementary motor area (pooled leads Cz, FCz′, FC1′, FC2′; [6], [36]). Lateralization of CNV over the motor area was also assessed (pooled leads C3, CP3′, C5′ minus C4, CP4′, C6′).

#### Motor potential (movement execution)

The lateralized initial motor potential peak was determined from 120 to 0 ms before the button press and represents a correlate of the sending of the command to muscle contraction from the primary motor cortex (pooled leads C3–C4, CP3′–CP4′, C5′–C6′ [9]).

#### Motor PINV (movement post-processing)

We analyzed lateralized movement-related post-processing (mPINV) over the motor area (pooled leads C3–C4, CP3′–CP4′, C5′–C6′) during the interval 400–800 ms after the unilateral index finger response movement, comparable to our previous studies [9], [14], [37], [38].

### Source analysis

Source analysis was carried out on the group grand averages, which provide the best signal-to-noise ratio. Dipole source modelling with equivalent dipoles allows the examination of group differences in the time course of event-related potential components. According to our previous study [9], we performed a source analysis by the automated RAP-MUSIC algorithm with SBSI on the motor PINV peak (400–600 ms after the response trigger). Two topographies were allowed according to the complexity of the scalp surface potential. Separate models were fit on the *DAT1* 6R–10R/6R–10R+*COMT* Met/Met (highest motor PINV amplitudes) and the *DAT1* other+*COMT* Val/Val group (low motor PINV amplitudes), because qualitative differences in motor PINV topography were found between the genetic groups.

In a second complementary approach with distributed sources instead of equivalent dipoles, sLORETA with the BESA default parameters (BESA GmbH, Munich, Germany) was performed on the same motor PINV time interval (non-lateralized data) in order to describe the extension of the distributed cortical sources of the lateralized motor PINV as exact as possible.

### Genotyping

EDTA anticoagulated venous blood samples were collected. Leukocyte genomic deoxyribonucleic acid (DNA) was isolated with the Qiamp DNA extraction kit (Qiagen, Chatsworth, California).

Genotyping of the *COMT* single nucleotide polymorphism (SNP) was completed using TaqMan (SNP) Genotyping Assays (7900HT Fast Real-Time-PCR-System; Applied Biosystems, Foster City, California). Amplification conditions for *COMT* rs4680 were: 3.0 µl TaqMan® Mastermix, 0.3 µl/0.15 µl TaqMan® oligonucleotide mix (20×/40×), 1.70 µl dH_2_O and 1 µl DNA solution (∼30 ng) in 96-well format in a 6 µl reaction. Amplification was performed by initial heating of 10 min–95°C, 40 cycles of 15 sec–95°C/1 min-60°C and final 10 min-4°C. TaqMan® assay-on-demand ID C_25746809_50 detected the alleles of rs4680 (hCV25746809) in the sequence context of CCAGCGGATGGTGGATTTCGCTGGC[A/G]TGAAGGACAAGGTGTGCATGCC.

The 40-bp VNTR polymorphism in the 3′-untranslated-region (UTR) of *DAT1* was genotyped with the primers and reaction conditions of Sano et al. [39]. Polymerase chain reaction was carried out using a nucleotide mix consisting of 7.4 mM deoxyadenosine triphosphate, deoxycytidine triphosphate, and deoxythymidine triphosphate and 3.7 mM deoxyguanosine triphosphate and 7-deaza-2′-deoxyguanosine 5′-triphosphate (Amersham Biosciences, Piscataway, NJ). After an initial denaturation step, 35 cycles of amplification of 1 minute at 94°C, 1 minute at 63°C, and 35 seconds at 72°C were performed. The 30-bp intron 8 VNTR polymorphism was genotyped according to the procedure by Vandenbergh et al. [40]. All genotypes were scored independently by 2 individuals who were blind to the presented data. The VNTRs had been genotyped in the context of the study of Laucht et al. [22]. No deviations from Hardy Weinberg equilibrium were detected (*DAT1* 30 bp VNTR intron 8 p = 0.78; *DAT1* 40 bp VNTR 3′UTR p = 0.10; *COMT* p = 0.20).

Both *DAT1* VNTRs were analyzed combined as haplotype. In accordance with the previous literature and in order to avoid small groups containing only a low number of subjects, with respect to the *DAT1* haplotype, subjects were dichotomized into homozygous carriers of the 6R–10R haplotype, which have previously been demonstrated to increase the risk of psychiatric disorders [22], and those who carried at least one non-risk haplotype.

In detail, the following genotype groups were formed: (1) *DAT1* haplotype: 6R–10R/6R–10R (N = 79) versus at least one non-6R–10R haplotype (N = 95); and (2) *COMT*: Val/Val (N = 43) versus Val/Met (N = 96) versus Met/Met (N = 35).

### Statistical analysis

To examine the effect of the *DAT1* haplotype (at least one non-6R–10R-haplotype was coded as ‘0’; 6R–10R/6R–10R was coded as ‘1’) and *COMT* (Val/Val = 0; Val/Met = 1; Met/Met = 2) on the target parameters (early CNV, late CNV, motor potential peak and lateralized motor PINV amplitudes), linear regression analyses were performed. In order to test for a significant epistasis between *DAT1* and *COMT*, regression models with and without an interaction term were compared. Significant interactions were further examined by conducting separate regression analyses for each *DAT1* haplotype level with *COMT* genotype as a predictor. All analyses included gender as a covariate. The same regression analysis as for EEG parameters was performed on behavioral measures, i.e. reaction time, reaction time variability, the number of omission and commission errors. In order to further examine the functional meaning of motor PINV, Pearson correlation coefficients were calculated between motor PINV amplitude and the behavioral measures.

## Results

### Behavioral data

Mean reaction time was 352±58 ms (± SD), with no significant effects of genetic variants. Trend level towards genetic influences on reaction time variability was not reached either (Table 1). There were on average 2.5±2.7 omission errors and 2.4±3.0 commission errors in the CPT task. While *DAT1* and *COMT* did not show any significant main effects or interactions on the number of commission errors, there was a significant interaction between the two functional polymorphisms with respect to omission errors (*DAT1* haplotype beta = −0.25; t = 1.8; p = 0.07; *COMT* beta = −0.16; t = 1.7; p = 0.098; *DAT1* haplotype x *COMT* beta = 0.31; t = 2.0; p = 0.047). However, separate regression analyses for the two *DAT1* haplotype groups with respect to *COMT* effects on omission errors did not yield significant results.

**Table 1:** Genetic influences on reaction times and reaction time variability (± standard deviation).

	Reaction time	RTSD^1^
**DAT1**		
6R–10R/6R–10R (N = 79)	353±63 ms	91±28 ms
at least one non-6R–10R allele (“DAT 1 other”; N = 95)	353±56 ms	89±31 ms
**COMT**		
Met/Met (N = 35)	359±65 ms	89±31 ms
Val/Met (N = 96)	349±60 ms	89±29 ms
Val/Val (N = 43)	359±53 ms	92±30 ms
**DAT1×COMT**		
6R–10R/6R–10R+Met/Met (N = 15)	346±52 ms	90±26 ms
6R–10R/6R–10R+Val/Met (N = 46)	354±66 ms	91±26 ms
6R–10R/6R–10R+Val/Val (N = 18)	359±68 ms	91±36 ms
DAT1 other+Met/Met (N = 20)	369±73 ms	89±35 ms
DAT1 other+Val/Met (N = 50)	344±54 ms	87±32 ms
DAT1 other+Val/Val (N = 25)	358±41 ms	93±26 ms

1 RTSD = intraindividual standard deviation of reaction times (reaction time variability).

### Event-related potential data

#### Effects of *DAT1* and *COMT* on orienting reaction, movement preparation and execution-related event-related potential components

**Figure 1:**
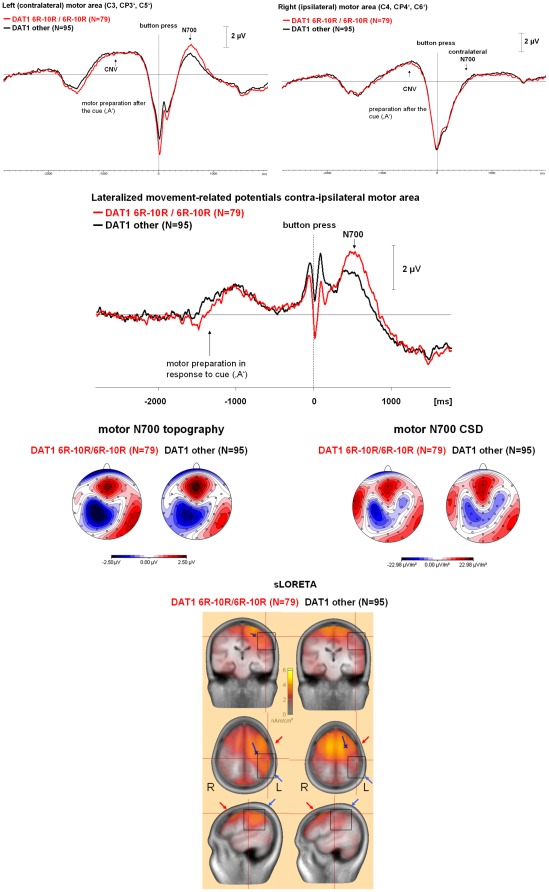
Time course and topography of the motor PINV by *DAT1* haplotype. **Top**: The time course of the response-locked motor PINV over the contra- and the ipsilateral motor area is shown. Negativity is up. There were no differences between the genotype groups during response preparation (contingent negative variation, CNV) after the cue (‘A’). Differences selectively affected the post-processing interval. During the button press (vertical dashed line), response selection during the P300 shadows the movement-related potentials. Thus, we calculated the lateralized motor PINV: Time course of the lateralized motor PINV when the potential over the contra- and ipsilateral motor areas is subtracted. This eliminates the symmetrically distributed parts of stimulus-related processing. Negativity is up. The peak immediately preceding the button press, which is related to the cortico-spinal command to muscle contraction, was influenced rather in the opposite direction to the motor PINV. **Middle**: Topography of the motor PINV: Isopotential line maps of the voltage topography and of the current source density (CSD) are shown, the head is presented in the top view from above, the nose is pointing upwards. Negativity and current sinks are reflected by blue areas, positivity and current sources are illustrated by red areas. Note the contralateral lateralization. **Bottom**: sLORETA source analysis results illustrating the effects of *DAT1* polymorphisms on the lateralized motor PINV: Note the stronger centro-parietal activation in Brodman areas 1–4 and 40 for the 6R–10R/6R–10R group, which is missing in the non 6R–10R/6R–10R group (marked by squares and blue arrows). Activation in the premotor area and frontal eye field (BA 6/8) was more bilateral in the non 6R–10R/6R–10R group (red arrows). The blue dipole indicates that RAP-MUSIC yielded a spatial component that showed a localization and orientation which explained the lateralized centro-parietal activation only for the 6R–10R/6R–10R group (details not shown). The crossing red lines were set to a point near the motor cortex hand area in order to illustrate the cortical activation in this area (cf. Figure 4).

Linear regression analyses revealed no significant impact of *DAT1*, *COMT* or their interaction on early and late CNV as well as on the initial motor potential peak for any examined target area (all p>0.10). Descriptive data pointed towards a non-significant *reduction* of lateralized negativity over the motor area during the initial motor potential peak for those genotypes which were associated with an increased lateralized motor PINV (cf. Figures 1–3).

**Figure 2 pone-0037814-g002:**
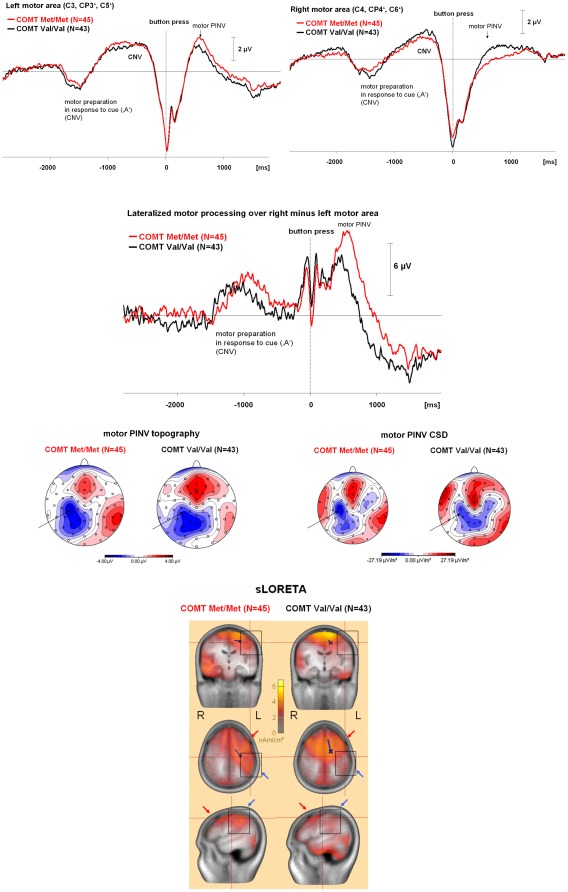
Time course and topography of the motor PINV by *COMT* Val^158^Met genotype. This Figure is organized as in Figure 1: **Top**: The time course of the response-locked motor PINV over the contra- and the ipsilateral motor areas is shown together with lateralized motor PINV. Negativity is up. There were no significant differences between the genotype groups during response preparation (contingent negative variation, CNV) after the cue (‘A’). The *COMT* genotype affected both contra- and ipsilateral potentials. These differences selectively affected the motor PINV interval. **Middle**: Topography of the motor PINV: Isopotential line maps of the voltage topography and of the current source density (CSD) are shown, the head is presented in the top view from above, the nose is pointing upwards. Negativity and current sinks are reflected by blue areas, positivity and current sources are illustrated by red areas. The arrows mark the contralateral motor area. **Bottom**: sLORETA source analysis results illustrating the effects of *COMT* polymorphisms on the lateralized motor PINV: Note the stronger centro-parietal activation for the Met/Met compared to the Val/Val group (marked by squares and blue arrows). However, also the frontal activity in Brodman areas 6/8 showed less lateralization in the Val/Val group (red arrows). The blue dipole indicates that RAP-MUSIC yielded a spatial component that showed a localization and orientation which explained the lateralized centro-parietal activation only for the Met/Met group (details not shown). The crossing red lines were set to a point near the motor cortex hand area in order to illustrate the cortical activation in this area (cf. Figure 4).

**Figure 3:**
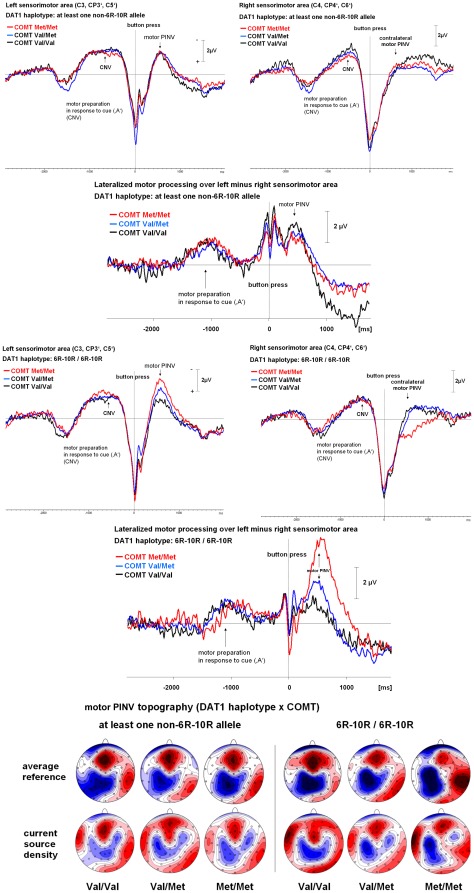
Time course and topography of the motor PINV – combined effects of *DAT1* and *COMT* genotypes. This Figure is organized as in Figures 1 and 2; however, the effects of the *COMT* genotype on the time course of the motor PINV are illustrated separately for the homozygous 6R–10R *DAT1* haplotype and for the *DAT1* haplotype with at least one non-6R–10R allele. Source analysis results are presented in Figure 4. **Top**: The time course of the response-locked motor PINV over the contra- and the ipsilateral motor area as well as their difference wave (lateralized motor PINV) is shown. Negativity is up. Influences of the *COMT* genotype for the *DAT1* haplotype with at least one non-6R–10R allele are shown. In this condition, there were no pronounced *COMT* effects on motor PINV. **Middle**: The time course of the response-locked motor PINV over the contra- and the ipsilateral motor area as well as their difference wave (lateralized motor PINV) is shown. Negativity is up. Influences of the *COMT* genotype for the homozygous 6R–10R *DAT1* haplotype are shown. In this condition, there was a strong effect of the *COMT* genotype on motor PINV. **Bottom**: Topography of the motor PINV: Isopotential line maps of the voltage topography and of the current source density (CSD) are shown, the head is presented in the top view from above, the nose is pointing upwards. Negativity and current sinks are reflected by blue areas, positivity and current sources are illustrated by red areas. Note that the Met/Met *COMT* genotype – especially in the presence of the homozygous 6R–10R *DAT1* haplotype – increased the lateralization of the topography of the motor PINV.

#### Lateralized motor PINV during response evaluation

The main finding of our study was that both *DAT1* and *COMT* significantly affected the lateralized motor PINV amplitude (F(4;169) = 4.1; p = 0.008; each p = 0.01 for DAT1 and COMT; linear regression model without an interaction term, see Table 2 for regression coefficients), with the amount of explained variance rising from 7 to 15% when an interaction term (p<0.0001) was included (F(4;169) = 7.3; p = 0.00002; see Table 2 for regression coefficients).

**Table 2:** Effects of DAT1 haplotype and COMT on the lateralized motor PINV – linear regression models^1^.

	B	SE B	beta	t-value	p
**without interaction (R^2^ = 0.07; p = 0.008)**					
Constant	−0.54±	0.64 µV			
Sex	−0.04±	0.36 µV	−0.008	0.1	n.s.
DAT1	−0.89±	0.36 µV	−0.19	2.5	0.01
COMT	−0.66±	0.27 µV	−0.18	2.5	0.01
**with interaction term DAT1×COMT (R^2^ = 0.15; p = 0.00002)**					
Constant	−1.49±	0.66 µV			
Sex	−0.01±	0.34 µV	−0.001	0.01	n.s.
DAT1	1.23±	0.64 µV	0.26	1.9	0.056
COMT	0.20±	0.34 µV	0.06	0.6	n.s.
DAT1×COMT	−2.03±	0.52 µV	−0.57	3.93	0.0001

1 B = regression coefficient; SE B = standard error of the regression coefficient; beta = standardized regression coefficient.

**Figure 4:**
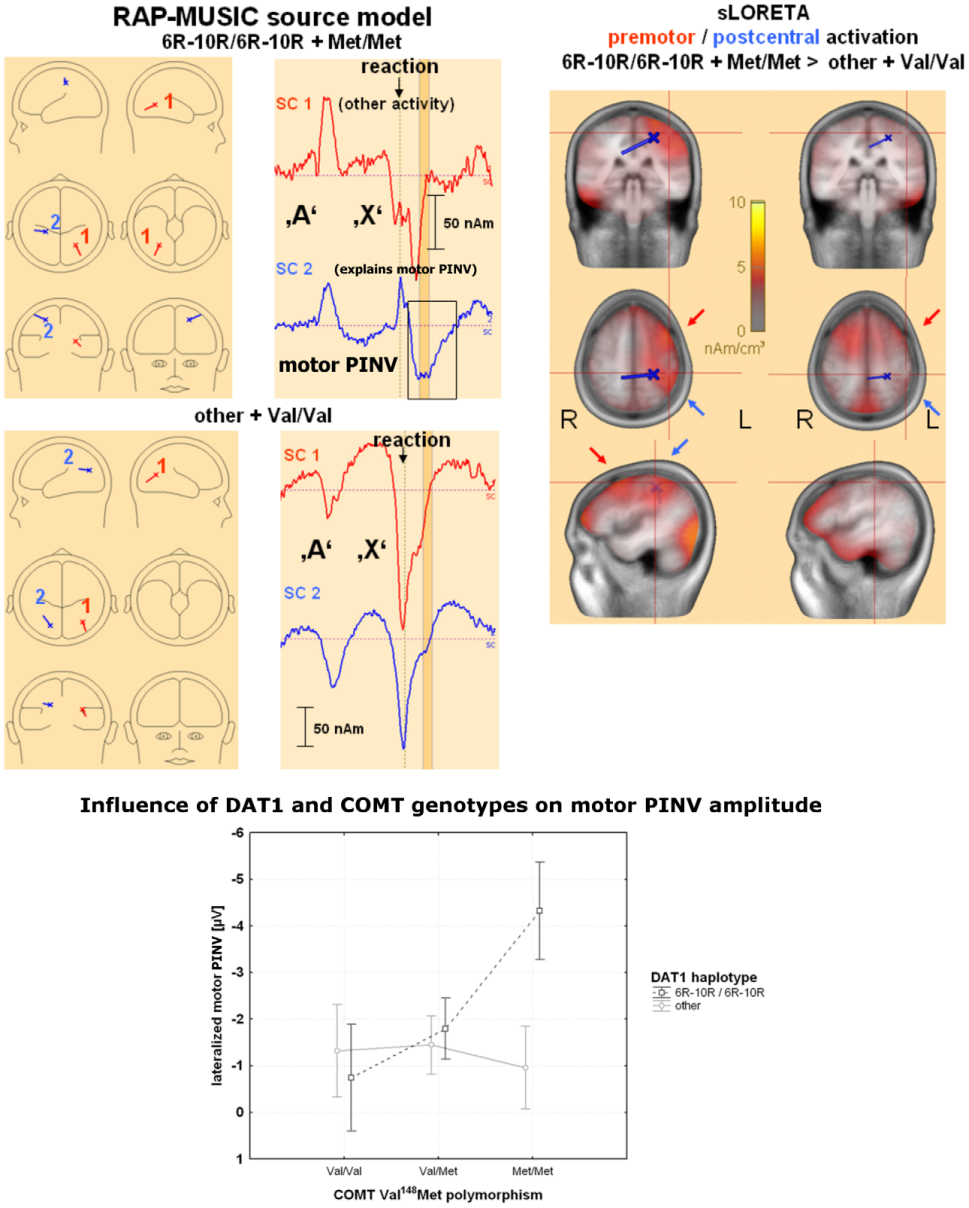
Combined effects of *DAT1* and *COMT* on lateralized motor PINV – source analysis. **A) Spatio-temporal dipole and sLORETA source analysis of influences of** ***COMT*** **and** ***DAT1*** **polymorphisms on lateralized motor PINV**. **Left**: RAP-MUSIC spatial component model fitted on the motor PINV peak. For the homozygous 6R–10R/Met group (highest lateralized motor PINV amplitudes), spatial component #2 explained left lateralized motor PINV topography, while spatial component #1 eliminates additional activity related to the visual post-processing and the P300/late positive complex. In contrast, for the homozygous other/Val group low lateralized motor PINV amplitudes, no comparable activation could be found. **Middle**: Dipole moments for the homozygous 6R–10R/Met group (highest lateralized motor PINV amplitudes) compared to the homozygous other/Val group (low motor PINV amplitudes). Colours and numbers refer to the models presented on the left. The vertical dashed line indicates the time of the button press trigger. The interval of the motor PINV peak (400–600 ms after the response) is marked in orange. **Right**: sLORETA source analysis for the same two genetic groups. The location of spatial component #2 for the homozygous 6R–10R/Met group depicted on the left is indicated (coordinates x = −0.30, y = −0.12, z = 0.54). The crossing red lines were moved to the point where this dipole projects onto the cortical surface in order to illustrate the cortical activation which was explained by this spatial component. Note that there were two areas with a stronger lateralized activation for the homozygous 6R–10R/Met group, one located more frontally around Brodman areas 6 and 8 (red arrow, premotor cortex and frontal eye field); the other located more centro-parietally comprising Brodman areas 1–4 and 40 (blue arrow, motor, somatosensory and posterior parietal cortex). **B) Interaction between the** ***DAT1*** **haplotype and** ***COMT*** **for the lateralized motor PINV**. The error bars indicate the 95% confidence intervals.

Figures 1 and 2 illustrate the separate effects of the *DAT1* haplotype (Figure 1) and the *COMT* (Figure 2) genotypes on the time course and topography of the motor PINV. The *DAT1* haplotype affected the centro-parietal negativity over the motor area located contralaterally to the response movement. In contrast, the *COMT* genotype was associated with the lateralization of the motor PINV and also showed effects on the ipsilateral hemisphere. Mean values and standard deviations of the lateralized motor PINV amplitudes are given in Table 3. Figure 3 illustrates the combined effects of *DAT1* and *COMT*, indicating that the *COMT* Met/Met genotype led to increased lateralized motor PINV amplitudes only in the presence of the homozygous 6R–10R *DAT1* haplotype. Figure 4 (source analysis) illustrates that the genetic effects (*DAT1*, *COMT* and their interaction) reflected differences in lateralized motor system activation (premotor cortex, primary motor and somatosensory cortex, posterior parietal cortex).

**Table 3:** Effects of DAT1 haplotype and COMT on the lateralized motor PINV – means and standard deviations.

Separate effects of DAT1 and COMT	Motor PINV amplitude [µV]
**DAT1 haplotype**	
6R–10R/6R–10R (N = 79)	−2.2±2.6 µV
Other (N = 95)	−1.3±2.1 µV
**COMT**	
Met/Met (N = 43)	−2.4±3.1 µV
Val/Met (N = 96)	−1.6±2.2 µV
Val/Val (N = 35)	−1.1±1.9 µV
**Combined effects of DAT1 and COMT**	
DAT1 10R-6R/10R-6R+COMT Met/Met (N = 18)	−4.3±2.9 µV
DAT1 other+COMT Met/Met (N = 25)	−0.95±2.3 µV
DAT1 10R-6R/10R-6R+COMT Val/Met (N = 46)	−1.8±2.2 µV
DAT1 other+COMT Val/Met (N = 50)	−1.4±2.2 µV
DAT1 10R-6R/10R-6R+COMT Val/Val (N = 15)	−0.74±2.1 µV
DAT1 other+COMT Val/Val (N = 20)	−1.3±1.8 µV

Separate linear regression analyses for the two *DAT1* haplotype groups (6R–10R/6R–10R and at least one non-6R–10R haplotype) indicated that the *COMT* genotype only had a significant effect on motor PINV amplitude in carriers of the homozygous 6R–10R *DAT1* haplotypes (R^2^ = 21%; regression slope −1.85±0.42 µV; beta = −0.46; t = −4.4; p = 0.00003) but not for carriers of other haplotype combinations (R^2^ = 1%; regression slope 0.21±0.32 µV; beta = 0.07; t = 0.7; n.s.). The largest lateralized motor PINV amplitudes occurred in those subjects carrying the Met/Met *COMT* polymorphism and being homozygous for the 6R–10R *DAT1* haplotype. The time course of the potential showed that the effects were specific for the motor PINV time interval because the early negative movement-related potentials around the button press trigger were unaffected by genetic variation.

In our sample, the degree of lateralization of the motor PINV was unrelated to differences in the degree of handedness between the subject groups: There was no correlation between the handedness index and motor PINV amplitude, as all six genetically defined groups (2 *DAT1* haplotype carrier groups ×3 *COMT* genotypes) presented mean handedness indexes ≥93. The highest handedness index was obtained for the group with at least one non-6R–10R and the Met/Met allele, i.e. the group that showed a smaller motor PINV lateralization than the 6R–10R/6R–10R+Met/Met group.

#### Relationships between behavioral parameters and lateralized motor PINV

There was a trend towards a negative correlation between mean reaction time and motor PINV lateralization (r = −0.14; t = 1.8; p = 0.066), indicating that more lateralized negative motor PINV amplitudes were associated with longer reaction times. Trend level for an association of larger reaction time variability and more lateralized negative motor PINV amplitudes was almost reached (r = −0.12; t = 1.6; p = 0.10).

Moreover, there was a significant correlation between lateralized motor PINV amplitude and the number of omission errors (r = −0.19; t = 2.5; p = 0.01), indicating that stronger motor post-processing during motor PINV on correct responses was associated with a higher number of omission errors. No such correlation of motor PINV with the number of commission errors was obtained (r = −0.04; t = 0.49; p = 0.63).

#### Source analysis of motor PINV

RAP-MUSIC: One spatial component (SC) near the central sulcus on the left hemisphere (contralateral to the response movement; blue spatial component #2 in Figure 4, top row) explained most of the left-sided centro-parietal scalp surface topography of the motor PINV. Another spatial component was used to eliminate additional activity related to the P300/late positive complex and visual influences (Figure 4, red spatial component #1). Residual variance during the motor PINV time interval was reduced to 4.8% by this simple model for the 6R–10R/6R–10R+Met/Met group showing the most pronounced lateralized motor PINV. The time-course of the dipole moment of spatial component #2 indicated transient lateralized motor post-processing. For the *DAT1* non 6R–10/6R–10R+*COMT* Val/Val group, no spatial component near the central sulcus was found (Figure 4, bottom row).

sLORETA revealed that activation in Brodman areas 6 and 8 (premotor cortex and frontal eye fields) was more lateralized in the subjects carrying the 6R–10R/6R–10R *DAT1* (Figure 1) and the Met/Met *COMT* genotypes (Figure 2), with stronger effects when the interaction between genes was examined (Figure 4). Contralateral sensorimotor post-processing (Brodman areas 1–4, 40) occurred only in the subjects carrying the 6R–10R/6R–10R *DAT1* and the Met/Met *COMT* genotypes (Figures 1, 2 and 4).

## Discussion

To the best of our knowledge, this is the first study to perform time-resolved genomic imaging of the influences of functional polymorphisms which are crucially involved in dopaminergic neurotransmission on orienting, movement preparation, execution and motor post-processing. Moreover, our study is the first to show an epistatic effect, i.e. an interaction between *DAT1* and *COMT*, which specifically affected the motor post-processing time interval. Interactions between these genes have been described so far by fMRI only with respect to other cognitive functions, namely reward sensitivity [41] and executive control [42].

In addition, a small effect with respect to an association of larger motor PINV amplitudes with longer reaction times and increased reaction time variability was observed. Moreover, larger motor PINV amplitudes were linked with more omission errors. These findings are consistent with those from another study [43] suggesting that distraction and less movement preparation may be associated with increased motor post-processing. A compensatory increased computational effort during motor post-processing for movements that are programmed less exactly seems plausible. In children, both the presence of the 6R–10R *DAT1* allele and increased distractibility (indexed by increased variability of reaction times and sometimes also increased mean reaction times) were demonstrated to be associated with the diagnosis of attention deficit hyperactivity disorder (ADHD) [22]. We found similar interactions between *DAT1* and *COMT* with regard to both the number of omission errors (an index of inattention) and lateralized motor PINV amplitude (which may be related to distraction as explained above).

The significant interaction between *DAT1* and *COMT* related to lateralized motor post-processing indicated that *COMT* exerted significantly stronger effects in homozygous carriers of the 6R–10R *DAT1* haplotype. In this case, individuals with the Met/Met genotype showed higher motor PINV amplitudes over contralateral motor areas than Val/Val carriers, while individuals with a heterozygous *COMT* exhibited intermediate motor PINV amplitudes.

With respect to motor PINV topography, 6R–10R/6R–10R haplotype and Met/Met genotype carriers showed a more focal response with a stronger motor PINV lateralization. sLORETA source analysis revealed that, during motor memory encoding, the extent of contralateral motor activation in the premotor cortex, primary motor cortex as well as posterior parietal cortex depended on *DAT1* and *COMT* genotypes. This activity was reflected in equivalent dipole source analysis by a spatial component near the central sulcus, which illustrated that this activation was specific for the post-processing interval (see dipole moments in Figure 4). There was no evidence for an increased processing during earlier movement execution.

Different movement stages (initiation, programming, execution and post-processing) involve different cortical and subcortical brain regions. While movement initiation involves the supplementary motor area and the basal ganglia [44], the programming of a movement is accomplished by the premotor and primary motor cortex [45] and relies on a reduction of the inhibition level in these areas [46], [47]. Though motor post-processing and movement-programming seem to involve the similar brain areas, the development of motor PINV amplitude during childhood and adolescence dissociates clearly from the development of movement-preparation related potentials [6], [35], indicating that both processes differ qualitatively and that different neuronal networks seem to be involved. While there is an increase of movement-preparation related negativity during childhood and adolescence, motor PINV shows the opposite development and a potential decrease during maturation. In children, movement-preparation related potentials are associated with a positive polarity, while motor PINV has a negative polarity [6], [35].

Thus, a selective influence of *DAT1* and *COMT* only on motor PINV without influences on preceding movement-related potentials is plausible as these potentials all reflect functionally different and separable processes, stages and networks. A differential modulation of motor PINV and contingent negative variation by dopamine antagonists (first generation antipsychotics) and in Parkinson's disease has been shown [14]. Our own data on the effects of methylphenidate on movement-related potentials [43] further support this dissociation of dopaminergic effects on pre- and post-movement potentials. A dopaminergic modulation of motor learning has been suggested [48] and may be important for post-processing and working memory encoding in the motor system and across different sensory modalities [37]. However, these hypotheses on why the motor PINV interval is specifically affected need to be addressed by further research.

Although no electromyographic data were available, movement-related potential lateralization is mostly independent of muscle force [49], [50], [51]. Our earlier findings in right-handed healthy adults showed that most of the signal with respect to the motor PINV component is contained in right-hand trials, as the lateralized motor PINV amplitude for button presses with the non-dominant left hand is small [9]. Moreover, the genetic influences in our sample on motor PINV lateralization were not mediated by differences in handedness either, as the Edinburgh Handedness Inventory scores followed a different pattern (cf. results section).

### A model of genetic influences on dopaminergic activity during stimulus post-processing

The *COMT* Met allele is likely to increase tonic dopamine levels and to reduce phasic dopamine responses [52]. As the Met and Val *COMT* alleles are co-dominant [18], heterozygous subjects should present an interim position between the homozygous individuals. COMT effects are mediated mainly via D1 dopamine receptors in the prefrontal cortex [10], [53]. *COMT* knockout mice show a prefrontal dopamine increase, with no similar effect in the striatum [54].

The explanation of why higher prefrontal (Met *COMT* allele) and lower striatal (10R *DAT1* allele) dopamine levels result in a more focused prefrontal cortical activation refers to the action via different types of dopamine receptors (D1/D2) and the nature of interactions in the cortico-striato-thalamo-cortical system [24]: While DAT1 effects take place in the striatum and are mediated by D2 receptors, COMT effects occur in the prefrontal cortex and are mediated by D1 receptors.

According to the tonic-phasic model [55], homozygous *COMT* Met allele carriers have an increased tonic dopamine level, but produce reduced phasic responses, with *COMT* Val allele carriers showing the inverse pattern [56]. Tonic prefrontal dopamine levels may be related to context maintenance, while phasic D2-receptor-related striatal dopamine levels may refer to working memory updating [57]. Prefrontal control can decrease striatal dopamine levels [58]. Our data may be best explained by an increase in dopamine concentrations in the prefrontal cortex (*COMT* Met-allele), which increases prefrontal cortex control of tonic dopamine level in the striatum [52], [58]. In the striatum, an increased *DAT1* expression induced by the 6R–10R haplotype [21] would also lead to decreased tonic dopamine levels, which produce larger phasic dopaminergic responses. When actions are followed by larger phasic striatal dopamine responses, this could lead to enhanced cortical motor post-processing, due to strong striato-cortical connections in the motor system. Phasic striatal dopamine responses could be elicited by movement execution. Dopaminergic action could continue during a movement post-processing period of about one or two seconds in order to facilitate an association of actions and their consequences and may differ from striatal contributions to movement initiation and preparation [59]. In our study, individuals with the homozygous *DAT1* 6R–10R haplotype and the *COMT* Met/Met genotype exhibited the largest motor PINV amplitudes. However, functional responses in the striatum (e.g. reward cue-related BOLD responses or dopamine release) have been found to be larger for carriers of the 9R-allele in some but not all studies [60], [61]. Thus, it is important to state that the exact mediating molecular mechanisms behind our findings still need to be disentangled in further studies.

At first glance, it seems highly plausible that genes which relate to dopamine inactivation affect stimulus post-processing. However, the finding that the post-processing *amplitude* rather than the duration of stimulus post-processing was mainly affected suggests that the effects were not directly related to the speed of dopamine inactivation by COMT or DAT1 [18]. Genetic effects were most likely mediated via influences on the level of tonic dopaminergic activity [52]. In this respect, motor system maturation must also be kept in mind, as, in children, the 6R–10R-allele has been described to be a risk factor for ADHD, while in adults the 6R–9R allele was associated with ADHD [62], [63], pointing towards a differential decay of dopamine transporter expression with increasing age [20]. Thus, our findings could be specific for healthy adolescents or even more specifically for healthy adolescents with biological and psychosocial risks for mental disorders, and may not simply generalize to adult subjects.

### Conclusions

Our findings suggest that tonic prefrontal and striatal dopamine levels interact. Specifically, in carriers of the 6R–10R/6R–10R+Met/Met genotype, a significant enhancement of motor post-processing could be shown in 15-year-old adolescents of a high-risk cohort. For the first time, dopaminergic genetic influences were observed on the time course of motor post-processing and were separated from preceding movement preparation and execution. Our results provide an example of how gene-gene interactions (apart from gene-environment interactions) can explain the limited effects of single genes on endophenotypes.
